# Meal timing trajectories in older adults and their associations with morbidity, genetic profiles, and mortality

**DOI:** 10.1038/s43856-025-01035-x

**Published:** 2025-09-04

**Authors:** Hassan S. Dashti, Chloe Liu, Hao Deng, Anushka Sharma, Antony Payton, Asri Maharani, Altug Didikoglu

**Affiliations:** 1 Department of Anesthesia, Critical Care and Pain Medicine, Massachusetts General Hospital, Boston, MA USA; 2 Division of Sleep Medicine, Harvard Medical School, Boston, MA USA; 3 Division of Nutrition, Harvard Medical School, Boston, MA USA; 4 Division of Informatics, Imaging & Data Sciences, School of Health Sciences, Faculty of Biology, Medicine and Health, The University of Manchester, Manchester, UK; 5 Division of Nursing, Midwifery and Social Work, School of Health Sciences, University of Manchester and Manchester Academic Health Science Centre (MAHSC), Manchester, UK; 6 Department of Neuroscience, Izmir Institute of Technology, Gulbahce, Urla, Izmir, Turkey; 7 Division of Neuroscience, School of Biological Sciences, Faculty of Biology, Medicine and Health, The University of Manchester, Manchester, UK

**Keywords:** Signs and symptoms, Diseases

## Abstract

**Background:**

Older adults are vulnerable to mistimed food intake due to health and environmental changes; characterizing meal timing may inform strategies to promote healthy aging. We investigated longitudinal trajectories of self-reported meal timing in older adults and their associations with morbidity, genetic profiles, and all-cause mortality.

**Methods:**

We analyzed data from 2945 community-dwelling older adults from the University of Manchester Longitudinal Study of Cognition in Normal Healthy Old Age, with up to five repeated assessments of meal timing and health behaviors conducted between 1983 and 2017. Linear mixed-effects models, latent class analysis, and Cox regression were used to examine relationships between meal timing with illness and behavioral factors, genetic scores for chronotype and obesity, and mortality.

**Results:**

Here we show older age is associated with later breakfast and dinner times, a later eating midpoint, and a shorter daily eating window. Physical and psychological illnesses, including fatigue, oral health problems, depression, anxiety, and multimorbidity, are primarily associated with later breakfast. Genetic profiles related to an evening chronotype, but not obesity, are linked to later meals. Later breakfast timing is also associated with increased mortality. Latent class analysis of meal timing trajectories identify early and late eating groups, with 10-year survival rates of 86.7% in the late eating group compared to 89.5% in the early eating group.

**Conclusions:**

Meal timing, particularly later breakfast, shifts with age and may reflect broader health changes in older adults, with implications for morbidity and longevity.

## Plain language summary

As people get older, changes in health and daily routines can affect when they eat their meals. This study followed nearly 3000 older adults in the UK over several decades to understand how meal timing changes with age and how it relates to health and longevity. Participants reported the times they ate meals and completed health and lifestyle surveys across multiple years. We found that as people aged, they tended to eat breakfast and dinner later, and those with more health problems or a genetic tendency to stay up late also tended to eat later. Importantly, eating breakfast later with aging was linked to a higher risk of death. Our findings suggest that later meal timing, especially breakfast, could serve as a simple marker of health in older adults and may guide future strategies for healthy aging.

## Abstract (web summary)

Dashti et al. characterize whether meal timing changes with age in older adults using longitudinal data from nearly 3000 individuals. They link later breakfast timing with increased multimorbidity and higher mortality risk and reveal behavioral and genetic factors that influence meal timing during aging.

## Introduction

Chrononutrition, the study of the timing of eating, has emerged as a modifiable risk factor for adverse health outcomes^1,2^. The relevance of the timing of dietary intake on health and mortality was initially demonstrated in animal and human studies in the late 1970s^3^. Recent studies in rodents indicating greater fat mass accumulation when high-fat diets are consumed during atypical periods (i.e., inactive phases) compared to typical times (i.e., active phases), have renewed interest in this area^4^. Subsequent human studies showed that later meal times may diminish the efficacy of behavioral weight loss interventions and that late eating is associated with metabolic changes leading to higher body mass index (BMI) and body fat accumulation^5–8^. In addition, experimental studies showed that endogenous melatonin in circulation in the evening impairs glucose tolerance^9^. The role of eating schedules is biologically relevant as dietary intake acts as an environmental cue influencing the circadian clocks of peripheral metabolic tissues and therefore can contribute to circadian misalignment and internal desynchrony^2,10^. The emerging evidence largely suggests that later meal times, particularly eating during the biological evening, is detrimental to health, and that eating at night is expected to be among the leading factors mediating the link between night shift work and higher risk of disease^2,11^. However, the role of chrononutrition in older adults remains underexplored^1,12^.

Older adults constitute a vulnerable population for mistimed food intake because of the onset of multimorbidity and the environmental changes that often accompany aging. Current chrononutrition studies in older adults are limited to cross-sectional analyses comparing older to younger adults^13–17^. Eating times are expected to shift in older adulthood, partly because of documented behavioral changes, such as earlier bed and wake times^18,19^. In addition, older adults are at risk of dysphagia, dehydration, impaired swallowing and declining oral health, all of which can negatively impact overall diet and increase the risk of malnutrition^20^. Transitioning to retirement or assisted living facilities can further affect nutritional status^21^. A key priority of the NIH Common Fund’s Nutrition for Precision Health, the NHLBI nutrition research priorities, and the NIH Sleep Research Plan is to advocate for meal times that align with the circadian system^1,22^. Carefully characterizing meal timing in older adults can inform relevant food timing countermeasures to support healthy aging.

No longitudinal study has yet described lifetime trajectories of mealtime patterns in older adults, and few studies have explored the broader factors influencing meal timing in this population^23^. Existing research on meal timing has disproportionately focused on younger healthy adults and night shift workers^2^. Key factors known to influence meal timing include intrinsic elements, such as medication use, sleep disorders, genetics, and chronotype, as well as extrinsic factors like cultural practices, environmental conditions, food availability, and work schedules^23^. This longitudinal analysis aims to characterize meal timing trajectories in older adults and investigate their associations with morbidity, behaviors, genetic profiles, and all-cause mortality, utilizing data from the University of Manchester Longitudinal Study of Cognition in Normal Healthy Old Age (UMLCHA) cohort.

The study shows that meal timing shifts with older age, particularly toward later breakfast and dinner, a delayed eating midpoint, and a shorter eating window. Later breakfast timing is associated with greater physical and psychological illness, multimorbidity, evening chronotype genetic profiles, and increased mortality risk. These findings suggest that shifts in meal timing may reflect underlying health and aging processes in older adults.

## Methods

### University of Manchester Longitudinal Study of Cognition in Normal Healthy Old Age

The University of Manchester Longitudinal Study of Cognition in Normal Healthy Old Age (UMLCHA) recruited 6375 individuals aged 42–94 years old from Newcastle and Manchester, UK, starting in 1983 through 1993^24^. All participants were followed until 2017, and therefore follow-up durations varying based on each participant’s enrollment date. Over the course of the study, participants were invited to complete optional questionnaires including those on sociodemographic, health status, and lifestyle^19^. Between 1999 and 2004, a subset of participants also provided whole blood samples. The study was approved by the University Research Ethics Committee at the University of Manchester (Ref: 2021-11274-17829), and all participants gave written informed consent prior to study procedures.

### Assessment of meal timing and sleep habits

The Personal Details Questionnaire, a cohort-specific survey that included questions on meal and sleep habits, sociodemographics, occupation, and subjective health, consistent with those used in other cohorts, was administered to participants up to five times. The initial administration occurred in person at enrollment, beginning in 1983 (baseline). The questionnaire was subsequently readministered to enrolled participants by mail at multiple points during the study: between 1984 and 1996 (second administration), 2001 and 2003 (third administration), 2007 (fourth administration), and 2010 (fifth and final administration). Due to variation in enrollment dates and follow-up participation, not all participants completed every questionnaire.

In the Personal Details Questionnaire, participants responded to questions pertaining to the timing of meals and sleep. Participants responded to the question, “What time do you eat [breakfast/lunch/dinner]?” In addition, participants responded to the questions, “Generally, at what time do you [go to bed at night/get up in the morning]?” and “On average, how many hours of sleep do you get every night?” Responses were used to calculate the time interval between waking and breakfast, the time interval between dinner and bed, the time interval between breakfast and dinner (i.e., daily eating window), and the midpoint between breakfast and dinner (i.e., eating midpoint).

### Mortality and other measures

Mortality data were obtained from the England NHS Digital death registry from the NHS England’s Secure Data Environment service for England (https://digital.nhs.uk/services/secure-data-environment-service). Access to the data was granted following approval of a data access request in adherence to the NHS Digital’s data governance and security protocols. The record data included information on the date of death up until August 2017.

At baseline, participants reported demographic information such as birth year, sex, socioeconomic status (based on the Standard Occupational Classification 2000), and education level (categorized using the International Standard Classification of Education 1997). On each of the five assessment occasions, participants also provided updates on their employment status (full-time, part-time, or unemployed/retired), smoking status, marital status, alcohol consumption, and subjective health status (rated on a scale from 1 (very bad) to 5 (very good)). Clinical measurements, including height and weight, were collected in-person for a subset of participants (*n* = 493 of the analytical sample). Through the repeated Personal Details Questionnaire, participants also reported daily cumulative time spent preparing meals (in hours) and whether they experienced any difficulty in meal preparation (yes/no). In addition, during their enrollment, participants completed the Pittsburgh Sleep Quality Index (PSQI) once and responses to the survey were used to compute scores reflecting sleep quality based on accepted thresholds^25^.

To assess symptoms of physical and psychological illness (i.e., morbidity), the Cornell Medical Index (CMI) was administered on four occasions, covering 19 general areas of symptomatology^26^. A participant was considered to have a health problem if they responded “yes” to any question corresponding to a specific health category within the CMI. A multimorbidity index was calculated by summing the number of health conditions reported by participants at each assessment.

### Polygenic scores

In a subset of participants, DNA was extracted from blood samples collected between 1999 and 2004, and genotyping was performed at the Wellcome Trust Clinical Research Facility Genetics Core, Western General Hospital, Edinburgh, using the Illumina610-Quadv1 chip (Illumina, San Diego, CA). Imputation was performed using the Haplotype Reference Consortium panel (GRCh37-hg19 genome assembly)^7^. Standard quality control was conducted: variants with >20% missing genotypes, variants not in Hardy-Weinberg Equilibrium, and individuals with a SNP call rate <80% were removed. Variants with frequency <0.1% were further excluded^24^. Population substructure correction was performed by calculating the principal components of ancestry using TRACE and the Human Genome Diversity Project^27,28^.

Genome-wide polygenic scores for eveningness (i.e., later chronotype) and obesity (i.e., higher BMI), scores with known associations with meal timing^29,30^, were calculated for each participant using PRS-CS (Polygenic Risk Score–Continuous Shrinkage), a Bayesian regression framework that places a continuous shrinkage prior on SNP effect sizes^31^. The UK Biobank EUR-ancestry linkage disequilibrium (LD) panel was used as the reference. The publicly available UK Biobank linkage disequilibrium matrices were obtained from Broad Institute’s Amazon repository (s3://broad-alkesgroup-ukbb-ld/UKBB_LD/). Effect estimates for the polygenic score were derived from the large genome-wide association study for evening preference in UK Biobank participants of European ancestry and the meta-analysis of genome-wide association studies for BMI in approximately 700,000 individuals of European ancestry by the Genetic Investigation of ANthropometric Traits (GIANT) consortium^32,33^. Each polygenic score was standardized to have a mean of 0 and a standard deviation of 1.

### Statistics and reproducibility

Participants who provided responses to all meal and sleep timing questions at baseline and had at least one additional meal timing response at any of the four subsequent occasions were included in the analysis. Descriptive statistics are presented as mean and standard deviations for continuous variables and frequency and percentages for categorical variables.

Changes in meal timing variables (dependent variables) with age (time-varying predictor) were assessed using linear mixed-effects models, as conducted previously^34^. An interaction term between age and wave was included to account for varying effects of age across different waves. Participant ID was also included as a random intercept to account for repeated measurements. The basic model (Model 1) adjusted for sex as a time-independent variable. The full model (Model 2) included additional time-independent variables such as socioeconomic status and education level, and time-dependent variables including sleep duration, employment status, smoking status, marital status, alcohol consumption, and subjective health status. Effects are reported per 10-years increase in age, with corresponding regression coefficients and 95% confidence intervals. For time-dependent variables with missing data in later cycles, values were carried forward once from previous cycles using the last observation carried forward approach. If still missing after the first carry-forward, the data were treated as missing (<5% missingness).

Latent class analysis was performed for meal timing to identify subgroups with distinct meal timing trajectories using LatentGOLD 5.1 (Statistical Innovations, Belmont, MA). The best-fitting model was determined by evaluating the average latent class posterior probability, Akaike Information Criterion (AIC) and Bayesian Information Criterion (BIC) with each added subgroup, ensuring that each subgroup included at least 5% of participants, and demonstrated meaningful distinction between subgroups (Supplementary Table 1). A two-cluster model was identified as the best fit. Meal timing variables were compared between the latent classes. The associations between the latent classes with the meal timing variables were also assessed using linear mixed-effects models as previously described.

The associations between physical and psychological illness (derived from the CMI) and meal-related behaviors (daily cumulative hours spent preparing meals and difficulty in meal preparation) with meal timing variables were analyzed using linear mixed-effects models, accounting for the covariates mentioned earlier with the inclusion of the age by wave interaction term in both models 1 and 2. Associations between PSQI score (sleep quality) with meal timing variables were analyzed using linear regression models, accounting for the same covariates. Linear mixed-effects models were also used to quantify the relationship between the scaled polygenic scores and meal timing variables accounting for the covariates mentioned above in the subset of 1226 participants with genetic data and 5 principal components of ancestry to account for population substructure. As confirmatory analyses, the polygenic score for evening chronotype was tested for associations with sleep midpoint, previously described as the midpoint between bed and wake times^19^, and the polygenic score for obesity was tested for associations with measured BMI. In sensitivity analyses, all model 1 analyses were further adjusted for the polygenic score for evening chronotype.

Meal timing trajectories and their associations with all-cause mortality were assessed using mixed-effects Cox regression models. For participants who died during follow-up, the time variable was defined as the period from recruitment to the date of death. For surviving participants, the time variable was defined as the period from recruitment to August 2017. The basic model (Model 1) only adjusted for sex as a time-independent variable and age as a time-dependent variable, whereas the full model (Model 2) additionally included time-independent variables such as socioeconomic status and education level, and time-dependent variables such as sleep duration, smoking status, and alcohol consumption. Results are presented as hazard ratios and 95% confidence intervals. For the meal timing clusters, Kaplan-Meier survival curves were generated, and log-rank tests were performed.

Associations with a two-sided *p* value < 0.05 were considered statistically significant. All analyses were two-tailed and performed using R version 4.3.1 (The R Foundation for Statistical Computing Platform, 2023) and the latest version of RStudio (Posit Software, PBC, Boston, MA).

### Reporting summary

Further information on research design is available in the Nature Portfolio Reporting Summary linked to this article.

## Results

### Meal timing trajectories across the older adult lifespan

A total of 2945 community-dwelling older adults recruited from Manchester and Newcastle, United Kingdom were included and had available self-reported meal timing data upon enrollment (baseline) and at least one additional repeat assessment (*n* = 2181 / 272 / 316 / 176 with 2 / 3 / 4 / 5 responses, respectively). At baseline, participants had a mean age of 64.0 years (standard deviation (SD) = 6.6; range = 42—94), 71.5% were female, and 83.3% were unemployed (Table 1). Participants reported average times for breakfast, lunch, and dinner of 8:22 (SD 0:43), 12:38 (SD 0:30), and 17:51 (SD 0:53), respectively. On average, participants had breakfast 31 min (SD 29) after waking up and had dinner 5.38 h (SD 1.07) before going to bed.

**Table 1:** Baseline characteristics of study participants (*N* = 2945)

	Mean (SD) or *n* (%)
Age at recruitment, years	64.0 (6.6)
Gender, female, *n* (%)	2107 (71.5)
Subjective health status, *n* (%)	
Very bad	10 (0.3)
Bad	65 (2.2)
Fair	608 (20.7)
Good	1460 (49.7)
Very good	792 (27.0)
Social class, professional occupations, *n* (%)	1219 (41.4)
Education level, *n* (%)	
Lower secondary education	1010 (34.3)
Upper secondary education	547 (18.6)
Post secondary non-tertiary education	647 (22.0)
Tertiary education first stage	720 (24.5)
Tertiary education second stage	17 (0.6)
Marital status, *n* (%)	
Single	270 (9.2)
Separated	169 (5.7)
Married	1832 (62.3)
Widowed	671 (22.8)
Employment status, *n* (%)	
Unemployed	2437 (83.3)
Part-time employment	314 (10.7)
Full-time employment	176 (6.0)
Smoking status, *n* (%)	
Never	1129 (38.5)
Past	1341 (45.8)
Current	461 (15.7)
Breakfast time, hh:mm	08:22 (0:43)
Lunch time, hh:mm	12:38 (0:30)
Dinner time, hh:mm	17:51 (0:53)
Interval from wake up to breakfast, hh:mm	0:31 (0:29)
Interval from dinner to bed, hh:mm	05:23 (1:04)

**Fig. 1:**
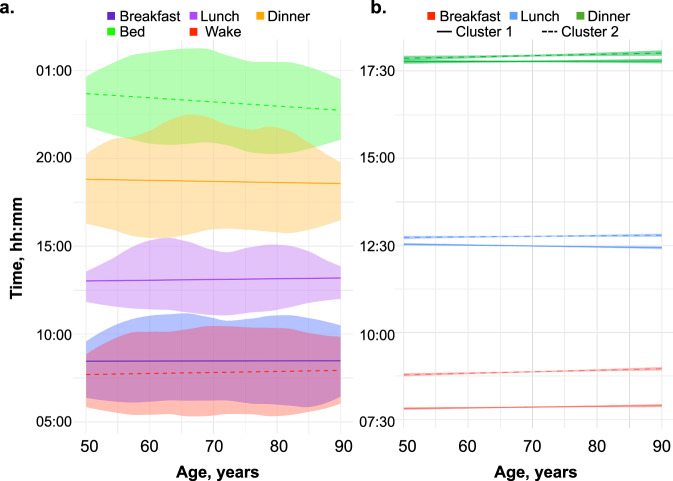
Trajectories in meal and bed times in older adults. Age-related changes in meal and bed times (**a**), and latent class differences in meal times and trajectory (**b**). The lines display model-predicted values from the linear mixed-effect models, with the shaded region indicating the 95% confidence interval of the estimates.

Older age was associated with later timing for breakfast in model 1 (Fig. 1a and Table 2). Each additional decade of aging was associated with a delay in breakfast of 7.94 min (95% CI: 5.56, 10.31). In model 2, the association remained significant, where each additional decade was associated with a delay in breakfast of 2.89 min (95% CI: 0.31, 5.46). Age trajectories were also evident for later midpoint of eating, shorter intervals from dinner to bed, and, in model 1 only, a shorter daily eating window. Lastly, an association with later dinner timing was observed only in model 2, where each additional decade of aging was associated with a delay in dinner of 3.67 min (95% CI: 0.6, 6.75).

**Table 2:** Age-related changes in meal timing (in minutes) in older adults per 10 years of aging

	Model 1		Model 2	
	Beta (95% CI)	*p* value	Beta (95% CI)	*p* value
Time of breakfast	7.94 (5.56, 10.31)	<0.001	2.89 (0.31, 5.46)	0.028
Time of lunch	−0.64 (−2.27, 0.99)	0.441	−0.35 (−2.19, 1.49)	0.710
Time of dinner	0.21 (−2.58, 3)	0.882	3.67 (0.6, 6.75)	0.019
Midpoint of eating	4.12 (1.85, 6.4)	<0.001	3.09 (0.53, 5.65)	0.018
Interval from wake up to breakfast	0.9 (−0.8, 2.59)	0.299	0.58 (−1.35, 2.51)	0.556
Interval from dinner to bed	−3.43 (−6.85, −0.01)	0.050	−8.98 (−12.77, −5.19)	<0.001
Daily eating window	−7.65 (4.2, 11.11)	<0.001	0.87 (−4.61, 2.87)	0.649

Results are presented as regression coefficients (95% confidence interval) in minutes and are shown per each 10 years of aging. Model 1 is only adjusted for sex as a time-independent variable; model 2 additionally included time-independent variables such as socioeconomic status and education level, and time-dependent variables such as age, sleep duration, employment status, smoking status, marital status, alcohol consumption, and subjective health status.

Latent class analysis of meal timing trajectories indicated heterogeneity in age-related changes in meal timing among older adults (Supplementary Tables 1 and 2). The analysis identified a two-cluster model as the best fit to capture the distinct meal timing patterns. The two clusters exhibited notable differences in their meal timing trajectories (Fig. 1b). The early eating subgroup (*n* = 1391) had consistently earlier meal times with aging, whereas the late eating subgroup (*n* = 1554) displayed generally later meal times with aging (Supplementary Table 3).

### Longitudinal associations of physical and psychological illness, behaviors, and genetic profiles with changes in meal timing

**Fig. 2:**
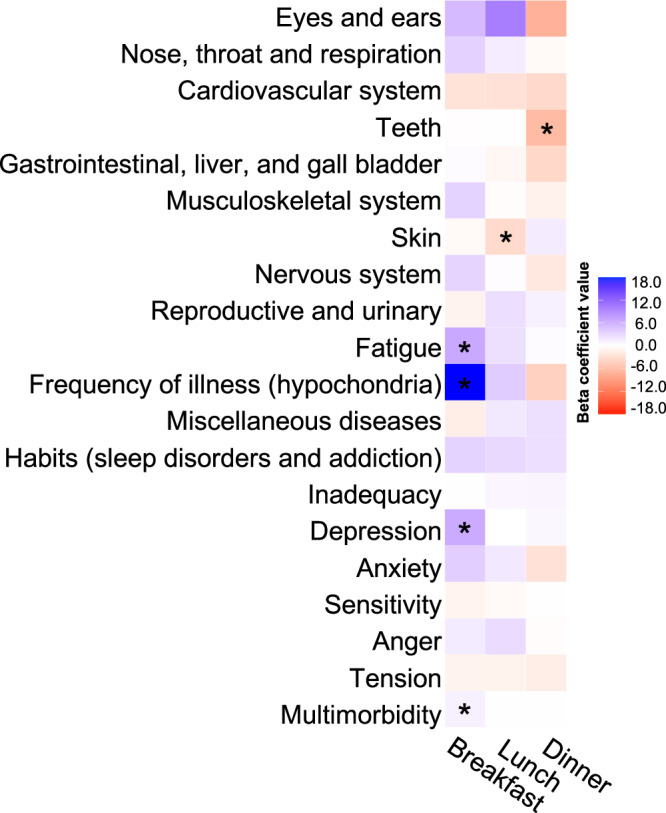
Heatmap of associations between physical and psychological illnesses and multimorbidity from the Cornell Medical Index with meal timing. Results are reported as regression coefficient (95% confidence interval) in minutes from model 1 adjusted for sex only. Positive coefficients indicate later meals; negative coefficients indicate earlier meals. Full association results are shown in tabular format in Supplementary Data 1.

To assess morbidity, participants were administered the Cornell Medical Index (CMI) on four occasions to report symptoms of physical and psychological illness^26^. Significant associations with meal timing were observed for both physical and psychological illnesses in linear mixed-effects models (Fig. 2, Supplementary Data 1 and 2). Among physical illnesses, associations were observed between having fatigue with both later breakfast timing and a shorter daily eating window. Having oral health problems was also linked to earlier dinner timing and a shorter daily eating window. Among psychological illnesses, associations were evident between depression and later breakfast time and a later eating midpoint, and between anxiety and a shorter daily eating window. Greater frequency of illness (i.e., hypochondria) was associated with later breakfast timing, a later eating midpoint, and a shorter eating window. Lastly, multimorbidity was also associated with later breakfast time, a later eating midpoint, and a shorter daily eating window. The associations remained largely unchanged after adjusting for lifestyle factors in model 2 (Supplementary Data 2) and the polygenic score for evening chronotype in sensitivity analysis (Supplementary Data 3).

Self-reported data on meal preparation behaviors and survey-derived sleep quality provided insight into additional lifestyle factors associated with meal timing trajectories. In linear mixed-effects models, longer meal preparation times were associated with a later dinner, a later eating midpoint, and a longer interval between waking and breakfast (model 2 only) (Supplementary Data 4). In addition, having difficulty preparing meals was associated with a later breakfast, a later eating midpoint, and a shorter daily eating window. In model 2, only the association with later breakfast remained significant. Lastly, higher PSQI score reflecting worse sleep quality was associated with a later breakfast and a shorter daily eating window in model 2 only (Supplementary Data 4).

**Fig. 3:**
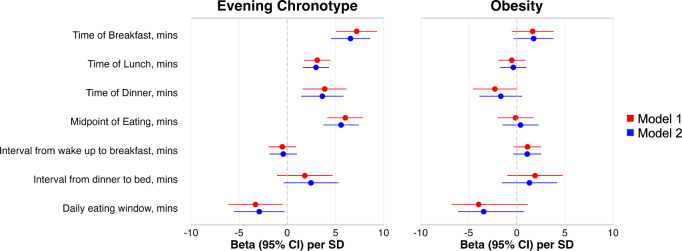
Evening chronotype and obesity polygenic scores associations with meal timing in older adults. Associations are adjusted beta values reflecting difference in meal timing in minutes per standard deviation of the polygenic scores. Model 1 is only adjusted for sex and 5 principal components of ancestry to account for population substructure as a time-independent variable and age as a time-dependent variable; model 2 additionally included time-independent variables such as socioeconomic status and education level, and time-dependent variables such as sleep duration, employment status, smoking status, marital status, alcohol consumption, and subjective health status. Association results are shown in tabular format in Supplementary Data 5.

To further identify determinants of meal timing, the role of polygenic scores for evening chronotype and obesity, which have previously been linked to eating times in younger populations^29,30^, were further examined in a subset of 1226 participants with genetic data. In confirmatory analyses, there were associations between polygenic scores for evening chronotype with later sleep midpoint (Beta (95% CI) = 6.65 (4.84, 8.47) minutes per SD) and for obesity with higher BMI (Beta (95% CI) = 1.54 (1.18, 1.91) kg/m^2^ per SD) (Supplementary Data 5). The polygenic score for evening chronotype was associated with later meals and eating midpoint (Fig. 3 and Supplementary Data 5). Specifically, each standard deviation increase in the polygenic score for eveningness was associated with later meal times: breakfast was later by 7.20 min (95% CI: 5.07, 9.34), lunch by 3.10 min (95% CI: 1.72, 4.47), and dinner by 3.88 min (95% CI: 1.61, 6.15). The polygenic score for evening chronotype and obesity were both associated with shorter daily eating windows.

### Meal timing and all-cause mortality

**Fig. 4:**
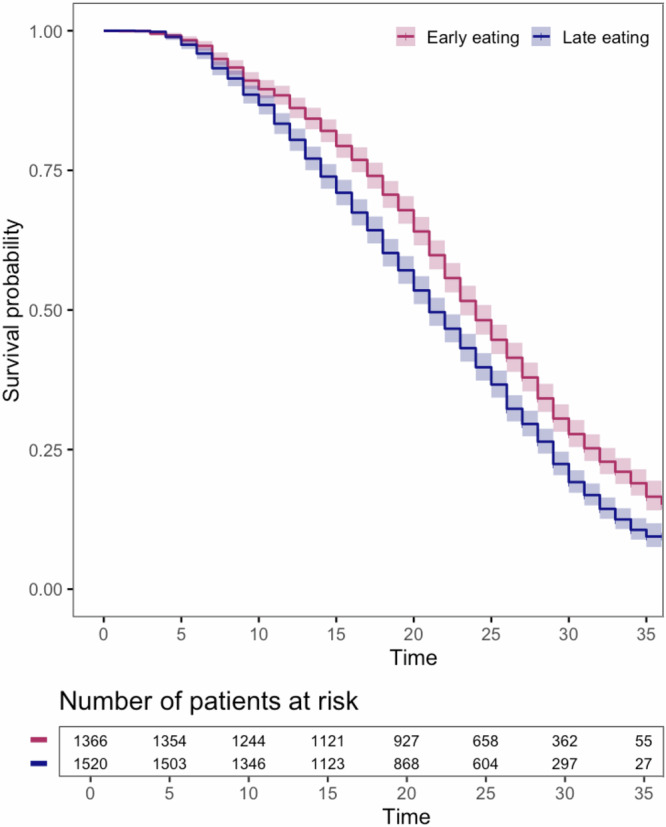
Kaplan–Meier survival curve for meal timing clusters and mortality. Kaplan–Meier survival estimates are shown as solid lines, with shaded regions representing the 95% confidence intervals at each time point.

During a mean follow-up of 22.0 years (SD 8.4), 2361 deaths were recorded over a total of 63,388 participant-years based on survival data from the National Health Service (NHS) Digital death register. The 10-year survival rate was 86.7% for the late group compared to 89.5% for the early group. Kaplan–Meier survival estimates indicated that individuals with later eating had a shorter survival compared to those with early eating (Fig. 4). Meal timing trajectories and their relationship with all-cause mortality were assessed using mixed-effects Cox regression models (Table 3 and Supplementary Table 4). Each additional hour of later breakfast timing was associated with a 1.11 (95% CI: 1.03, 1.18) increase in odds of mortality in model 1, and a 1.08 (95% CI: 1.00, 1.17) increase in model 2 (Table 3).

**Table 3:** Associations between meal timing (in hours) and mortality using mixed-effects Cox regression models

	Model 1		Model 2	
	Hazard ratio (95% CI)	*p* value	Hazard ratio (95% CI)	*p* value
Time of breakfast	1.11 (1.03, 1.18)	0.010	1.08 (1, 1.17)	0.050
Time of lunch	1.05 (0.95, 1.15)	0.300	1.05 (0.94, 1.15)	0.390
Time of dinner	1.03 (0.97, 1.09)	0.350	1.05 (0.99, 1.12)	0.120
Midpoint of eating	1.06 (0.99, 1.13)	0.084	1.07 (1, 1.15)	0.052
Interval from wake up to breakfast	1.05 (0.96, 1.14)	0.310	1.05 (0.95, 1.15)	0.340
Interval from dinner to bed	0.98 (0.93, 1.03)	0.450	0.76 (−1.96, 1.96)	>0.999
Daily eating window	0.97 (0.93, 1.02)	0.410	1.00 (0.95, 1.05)	0.910

Results are presented as hazard ratios and 95% confidence intervals. Model 1 only adjusted for sex as a time-independent variable and age as a time-dependent variable, whereas Model 2 additionally included time-independent variables such as socioeconomic status and education level, and time-dependent variables such as sleep duration, smoking status, and alcohol consumption.

## Discussion

Longitudinal data from a large cohort of community-dwelling older adults, followed for over 20 years, showed gradual shifts in meal timing with aging, specifically toward later breakfast, dinner, and eating midpoint and a shorter interval between dinner and sleep. The onset of physical and psychological illnesses including multimorbidity, challenges with meal preparation and worse sleep, and genetic predispositions partly contributed to the observed shifts in meal timing. Distinct meal timing trajectories, particularly those characterized by later breakfast, were associated with increased mortality risk.

There is growing evidence to suggest that eating behaviors, including eating times, shift with aging^13–17^. Few cross-sectional studies compared meal timing in older and younger adults, although with mixed results. For example, data from the National Health and Nutrition Examination Survey (NHANES) suggest that older adults skip breakfast less frequently and have a later eating midpoint compared to younger individuals^14^. Conversely, other studies from the US and Japan report earlier meal times in older adults compared to younger adults^16,17^. The present study extends these findings by providing longitudinal data with repeat assessments of meal timing showing that meal timing continues to change throughout older adulthood. Specifically, the data suggest later breakfast and dinner times and a later eating midpoint with age, while lunch timing remains stable likely due to limited variability in lunch schedules across participants^35^. Although the per-decade shifts in meal timing were modest, they accumulate to a more substantial difference (approximately 45 min for breakfast timing) when considered across the full age range of participants spanning over five decades. The concurrent assessment of bed and wake times with the timing of traditional meals allowed for other relevant meal timing measures to be examined in this study, such as the interval from wake-up to breakfast and from dinner to bedtime. Consistent with prior studies, the data suggest shorter intervals between dinner and bed with aging independent of sleep changes^16^.

Morbidity may contribute to changes in meal timing among older adults. The systematic analysis of 19 illnesses indicated notable associations for depression, anxiety, and fatigue, and declining oral health with later meals. Furthermore, multimorbidity is also associated with later eating. Late eating in younger and older adults has previously been implicated in obesity and other metabolic disorders such as diabetes^8,15,36^. Limited evidence also suggests that meal timing also impacts physical health in older adults. For example, in a larger observational study of older adults, later eating was associated with lower grip strength, and a shorter eating interval was associated with lower muscle mass^12^. In another study, short-term time-restricted eating in older adults did not lead to physical or mental fatigue^37^. Although the observational design cannot establish directionality, it is more likely that the onset of disease leads to shifts in meal timing rather than shifts in meal timing contributing to the onset of disease. Overall, the results suggest that age-related physical and psychological health factors, including worse sleep, may play a role in shaping meal timing in aging populations, with multimorbidity potentially exacerbating these changes.

Genetic profiles for evening chronotype and obesity have been previously implicated with meal timing including later breakfast and breakfast skipping^29,30,38^. The present findings are consistent with findings in younger adults, demonstrating robust associations between evening chronotype genetic profiles and later meal times. The genetic analyses also corroborates epidemiological data indicating later eating among evening-type older adults^39^. The findings do not suggest that obesity-implicated genetic variants that are known to influence eating behaviors such as satiety and taste preferences further influence eating times in older adults^29^. Associations between both evening chronotype and obesity genetic risks with shorter eating windows may indicate post-meal snacking behavior among individuals at higher genetic risk. Overall, these findings suggest that individuals with genetic predispositions toward evening chronotype may be at the highest risk for late eating behaviors.

In the present study, later breakfast is associated with an increased risk of mortality, although the association was attenuated when considering the effects of important confounders. This finding is consistent with a previous study showing associations between breakfast skipping and increased mortality in older adults^40^. No associations with mortality were evident for the timing of lunch or dinner, length of the eating window, or the proximity of meals to sleep. In addition, the late eating cluster was associated with a lower survival rate compared to the early eating cluster. Age-related changes in the microbiome are known to influence energy utilization and appetite, which may partially mediate the observed associations^41^.

Anorexia of aging is a prevalent geriatric syndrome, characterized by a loss of appetite and reduced food intake, and is associated with undernutrition^42,43^. Established causes of anorexia of aging are changes in oral health, alterations in chemosensory and gastrointestinal functions such as swallowing disorders, cognitive decline and dementia, social factors, and medication side effects that may alter taste and smell or induce dryness and constipation^42,43^. The present findings suggest that the onset of anorexia of aging may be also accompanied by shifts toward later meal times, particularly breakfast. However, given the modest effect sizes observed, established screening approaches for anorexia of aging, such as regular weight monitoring and dietary assessment, remain essential for tracking overall health decline in older adults. Aging is known to dampen the entraining effects of behavioral cues, such as meal timing, thereby reducing the sensitivity of peripheral tissues to nutrients^44^. Based on the present findings, gradual delays in meal timing with age may mediate the observed effect. The results support the hypothesis that maintaining consistent eating schedules, including breakfast times, could restore circadian rhythms in older adults^45^. Addressing the onset of pathologies such as depression and fatigue that may lead to meal delays may support robust rhythms in older adults.

A major strength of this study is the repeated assessment of meal timing in a large cohort of community-dwelling older adults (though most participants had only two assessments given the aging nature of this older adult cohort) allowing for the consideration of both inter- and intra-individual variation in meal timing patterns.

Additionally, the concurrent assessment of sleep and relevant sociodemographic factors allowed for the inclusion of other meal timing variables in the longitudinal analyses. However, several limitations should be noted. Currently, there are no standard methodologies or established variables for chrononutrition, making it difficult to compare results across studies and cohorts^46^. Furthermore, the study was unable to derive other common meal timing variables, such as eating frequency, meal timing variability, and the first and last eating occasions, which may capture snack consumption outside traditional meal times^12^. However, previous studies using 24-h recalls in older adults indicate that most tend to consume three primary meals, justifying the focus on the traditional meals in the analysis^47^. In addition, while some research suggests considerable day-to-day variation in meal timing among older adults^48^, others have not^17^. Therefore, the questions on mealtimes and bedtime routines may not accurately reflect participants’ actual behavioral patterns, and assumptions about typical eating habits based on other populations may not apply to the present sample. Additionally, meal frequency and skipping behaviors, areas of interest in older adults and chrononutrition, were not examined as missing meal timing data could have been misinterpreted as skipped meals. Moreover, the timing of specific nutrients, including protein content, which has been shown to associate with skeletal muscle mass in older adults when ingested before sleep, was not evaluated in this study^49,50^. Another limitation is the reliance on the CMI for capturing morbidity, which, although correlated with clinical diagnoses, may not identify asymptomatic conditions that could only be detected through laboratory tests or physical examinations^51^. The cohort recruited healthy, community-dwelling older adults of British European ancestry, which may limit the generalizability of the findings. Non-random attrition and dropout effects in this older adult population may have introduced bias because participants with poorer health may have fewer repeated assessments^52^. Lastly, although our models accounted for several potential confounders, they did not include physical activity, and residual confounding remains possible.

In summary, the findings suggest that meal timing shifts with aging and is associated with morbidity and mortality in older adults. Future research, including randomized controlled trials, is needed to explore the potential of meal timing as a strategy to promote longevity in aging populations.

## Supplementary information

Supplementary Information

Description of Additional Supplementary Files

Supplementary Data 1

Supplementary Data 2

Supplementary Data 3

Supplementary Data 4

Supplementary Data 5

Reporting Summary

## Data Availability

The data used in this manuscript from the Manchester Longitudinal Cognitive Ageing Cohort are not publicly available. However, they may be shared upon reasonable request and the completion of a Material Transfer Agreement. England NHS Digital death registry data used in this study are available in NHS England’s Secure Data Environment service for England, but as restrictions apply are not publicly available (https://digital.nhs.uk/services/secure-data-environment-service). Access to the NHS registry data can be requested by completing the Data Access Request application form via https://dataaccessrequest.hscic.gov.uk/. UK Biobank linkage disequilibrium matrices are publicly available at s3://broad-alkesgroup-ukbb-ld/UKBB_LD/. The Source data for Fig. 2 is in Supplementary Data 1 and the Source data for Fig. 3 is in Supplementary Data 5.

## Code availability

The code used in this manuscript from the Manchester Longitudinal Cognitive Ageing Cohort are not publicly available. However, they may be shared upon reasonable request and the completion of a Material Transfer Agreement.
