# Supplementary material for: Meal timing trajectories in older adults and their associations with morbidity, genetic profiles, and mortality
Source: Commun Med (Lond). 2025 Sep 4;5:385. doi: 10.1038/s43856-025-01035-x (PMC12411609; doi:10.1038/s43856-025-01035-x)
Supplement: Supplementary file 1 — Supplementary Information [file 43856_2025_1035_MOESM1_ESM.pdf]

Supplementary Table 1. Evaluating latent classes for meal times (2 – 4 class models)

Models		2 class	3 class	4 class
Log-likelihood Statistics	LL	-7555.87	-7166.03	-7020.94
	AIC	15121.75	14346.05	14059.88
	BIC	15151.68	14387.97	14113.77
	SABIC	15135.80	14365.73	14085.18
	CAIC	15156.68	14394.97	14122.77
Classification Statistics	Smallest class count	1391.00	395.00	116.00
	Smallest class size (%)	47.23	13.41	3.94
	Classification errors	0.13	0.12	0.18
	Entropy R-squared	0.56	0.67	0.63
	Standard R-squared	0.62	0.66	0.59
	Classification log-likelihood	-8444.06	-7998.48	-8232.88
	Entropy	888.19	832.45	1211.94
	CLC	16888.12	15996.95	16465.76
	AWE	16982.99	16129.78	16636.54
	ICL-BIC	16928.05	16052.87	16537.65
	ALCPP	0.89	0.88	0.82
		0.86	0.91	0.91
			0.82	0.90
				0.82

N participant = 2945, number of observations = 7288. LL = log-likelihood; AIC = Akaike information criterion; BIC = Bayesian information criterion; SABIC = sample-size adjusted BIC; CAIC = consistent Akaike information criterion; CLC = classification likelihood criterion; AWE = approximate weight of evidence criterion; ICL-BIC = integrated classification likelihood (BIC approximation); ALCPP = average latent class posterior probability. Changes of breakfast time across age (years) are shown in the graphs below for each latent class model.

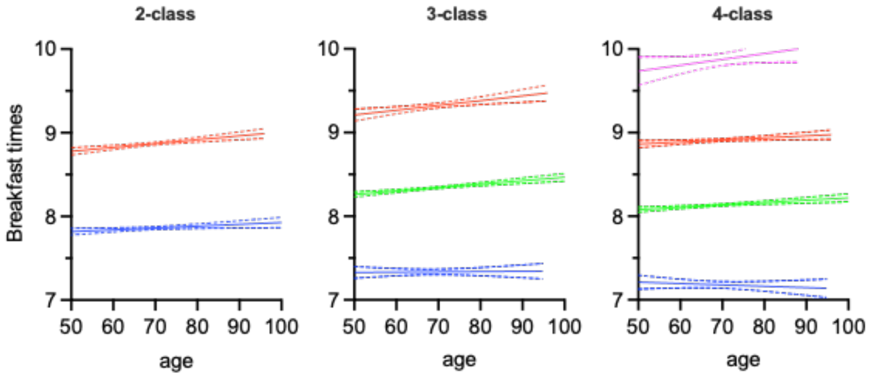

Supplementary Table 2. Baseline characteristics of study participants stratified by early (*n* = 1,391) and late (*n* = 1,554) eating clusters.

	Cluster 1 - Early Eating	Cluster 2 - Late Eating	*P* value
	Mean (SD) or *n* (%)	Mean (SD) or *n* (%)	
Age at recruitment, years	63.3 (6.9)	64.7 (6.2)	<0.001
Gender, female, *n* (%)	996 (71.6)	1,111 (71.5)	0.980
Subjective health status, *n* (%)			<0.001
Very bad	2 (0.1)	8 (0.5)	
Bad	19 (1.4)	46 (3.0)	
Fair	247 (17.8)	361 (23.3)	
Good	692 (49.9)	768 (49.6)	
Very good	428 (30.8)	364 (23.5)	
Social class, professional occupations, *n* (%)	607 (43.7)	612 (39.4)	0.020
Education level, *n* (%)			0.002
Lower secondary education	433 (31.2)	577 (37.1)	
Upper secondary education	264 (19.0)	283 (18.2)	
Post secondary non-tertiary education	301 (21.7)	346 (22.3)	
Tertiary education first stage	380 (27.4)	340 (21.9)	
Tertiary education second stage	9 (0.6)	8 (0.5)	
Marital status, *n* (%)			0.639
Single	127 (9.1)	143 (9.2)	
Separated	88 (6.3)	81 (5.2)	
Married	863 (62.0)	969 (62.5)	
Widowed	313 (22.5)	358 (23.1)	
Employment status, *n* (%)			<0.001
Unemployed	1,077 (78.2)	1,360 (87.8)	
Part-time employment	171 (12.4)	143 (9.2)	
Full-time employment	130 (9.4)	46 (3.0)	
Smoking status, *n* (%)			<0.001
Never	579 (41.8)	550 (35.6)	
Past	629 (45.4)	712 (46.1)	
Current	178 (12.8)	283 (18.3)	
Breakfast time, hh:mm	07:51 (00:31)	08:50 (00:32)	<0.001
Lunch time, hh:mm	12:30 (00:28)	12:45 (00:31)	<0.001
Dinner time, hh:mm	17:47 (00:51)	17:56 (00:55)	<0.001
Interval from wake up to breakfast, hh:mm	00:26 (00:28)	00:35 (00:30)	<0.001
Interval from dinner to bed, hh:mm	05:17 (01:01)	05:29 (01:06)	<0.001

Supplementary Table 3. Effects of age on meal timing (in minutes) in older adults by clusters.

	Cluster 1 (*n* = 1,391) - Early Eating					Cluster 2 (*n* = 1,554) - Late Eating				
	Baseline in hh:mm	Model 1		Model 2		Baseline in hh:mm	Model 1		Model 2	
	Mean (SD)	Beta (95% CI)	*P* value	Beta (95% CI)	*P* value	Mean (SD)	Beta (95% CI)	*P* value	Beta (95% CI)	*P* value
Time of breakfast	07:51 (00:31)	3.9 (1.46, 6.35)	0.002	-0.45 (-3.31, 2.41)	0.758	08:50 (00:32)	2.91 (0.27, 5.54)	0.031	0.38 (-2.5, 3.26)	0.795
Time of lunch	12:30 (00:28)	-2.49 (-4.55, -0.43)	0.018	-1.21 (-3.61, 1.2)	0.325	12:45 (00:31)	-0.94 (-3.36, 1.49)	0.450	-0.53 (-3.17, 2.11)	0.694
Time of dinner	17:47 (00:51)	-2.3 (-5.98, 1.39)	0.222	2.37 (-1.81, 6.55)	0.266	17:56 (00:55)	1.51 (-2.71, 5.73)	0.485	3.77 (-0.76, 8.31)	0.103
Midpoint of eating	12:49 (00:30)	1.15 (-1.78, 4.08)	0.440	1.14 (-2.31, 4.59)	0.516	13:23 (00:34)	2.07 (-0.93, 5.08)	0.176	2.32 (-0.89, 5.53)	0.157
Interval from wake up to breakfast	00:26 (00:28)	1.2 (-1.09, 3.49)	0.304	1.19 (-1.51, 3.88)	0.388	00:35 (00:30)	-0.84 (-3.32, 1.63)	0.504	-1.56 (-4.25, 1.14)	0.257
Interval from dinner to bed	05:17 (01:01)	-1.93 (-6.49, 2.64)	0.409	-8.53 (-13.76, -3.3)	0.001	05:29 (01:06)	-6.98 (-12.1, -1.85)	0.008	-11.04 (-16.53, -5.55)	<0.001
Daily eating window	09:56 (00:59)	-5.94 (-10.25, -1.62)	0.007	3.87 (-1.03, 8.76)	0.122	09:07 (01:01)	-1.73 (-6.49, 3.03)	0.476	3.29 (-1.8, 8.38)	0.205

Results are presented as regression coefficients (95% confidence interval) and are shown per each 10 years of aging. Model 1 is only adjusted for sex as a time-independent variable and age as a time-dependent variables; model 2 additionally included time-independent variables such as socioeconomic status and education level, and time-dependent variables such as sleep duration, employment status, smoking status, marital status, alcohol consumption, and subjective health status.

Supplementary Table 4. Sensitivity analyses further adjusting for the polygenic score for evening chronotype for the associations between meal timing (in hours) and mortality using mixed-effects Cox regression models.

	Model 1 + polygenic score for evening chronotype	
	Hazard ratio (95% CI)	*P* value
Time of breakfast	1.05 (0.96, 1.15)	0.270
Time of lunch	1.04 (0.91, 1.17)	0.540
Time of dinner	1.03 (0.94, 1.12)	0.530
Midpoint of eating	1.03 (0.94, 1.12)	0.510
Interval from wake up to breakfast	1.05 (0.92, 1.17)	0.470
Interval from dinner to bed	0.94 (-4.16, 4.17)	1.000
Daily eating window	0.85 (-2.86, 2.86)	1.000
