# Supplementary material for: Meal timing trajectories in older adults and their associations with morbidity, genetic profiles, and mortality
Source: Commun Med (Lond). 2025 Sep 4;5:385. doi: 10.1038/s43856-025-01035-x (PMC12411609; doi:10.1038/s43856-025-01035-x)
Supplement: Supplementary file 2 — Description of Additional Supplementary Files [file 43856_2025_1035_MOESM2_ESM.pdf]

Description of Additional Supplementary Files

File name- Supplementary Data 1

File description – Association results are reported as regression coefficient (95% confidence interval). Model 1 is only adjusted for sex as a time-independent variable; model 2 additionally included time-independent variables such as socioeconomic status and education level, and time-dependent variables such as age, sleep duration, employment status, smoking status, marital status, alcohol consumption, and subjective health status.

File name- Supplementary Data 2

File description – Model 2 associations between 19 physical and psychological illnesses and multimorbidity derived from the Cornell Medical Index with meal timing.

File name- Supplementary Data 3

File description – Sensitivity analyses further adjusting for the polygenic score for evening chronotype for the associations between meal timing (in hours) and 19 physical and psychological illnesses and multimorbidity

File name- Supplementary Data 4

File description – Associations between self-reported meal preparation behaviors and sleep quality with meal timing in older adults.

File name- Supplementary Data 5

File description – Associations between the polygenic score for evening chronotype and obesity with BMI and sleep midpoint (confirmatory analyses) and meal timing in older adults.
