# Supplementary material for: Meal timing trajectories in older adults and their associations with morbidity, genetic profiles, and mortality
Source: Commun Med (Lond). 2025 Sep 4;5:385. doi: 10.1038/s43856-025-01035-x (PMC12411609; doi:10.1038/s43856-025-01035-x)
Supplement: Supplementary file 8 — Reporting Summary [file 43856_2025_1035_MOESM8_ESM.pdf]

nature portfolio

Corresponding author(s): Hassan S Dashti

Last updated by author(s): Feb 25, 2025

Reporting Summary

Nature Portfolio wishes to improve the reproducibility of the work that we publish. This form provides structure for consistency and transparency in reporting. For further information on Nature Portfolio policies, see our Editorial Policies and the Editorial Policy Checklist.

Statistics

For all statistical analyses, confirm that the following items are present in the figure legend, table legend, main text, or Methods section.

n/a	Confirmed	
☐	☒	The exact sample size (n) for each experimental group/condition, given as a discrete number and unit of measurement
☐	☒	A statement on whether measurements were taken from distinct samples or whether the same sample was measured repeatedly
☐	☒	The statistical test(s) used AND whether they are one- or two-sided<br>*Only common tests should be described solely by name; describe more complex techniques in the Methods section.*
☐	☒	A description of all covariates tested
☐	☒	A description of any assumptions or corrections, such as tests of normality and adjustment for multiple comparisons
☐	☒	A full description of the statistical parameters including central tendency (e.g. means) or other basic estimates (e.g. regression coefficient) AND variation (e.g. standard deviation) or associated estimates of uncertainty (e.g. confidence intervals)
☒	☐	For null hypothesis testing, the test statistic (e.g. F, t, r) with confidence intervals, effect sizes, degrees of freedom and P value noted<br>*Give P values as exact values whenever suitable.*
☒	☐	For Bayesian analysis, information on the choice of priors and Markov chain Monte Carlo settings
☒	☐	For hierarchical and complex designs, identification of the appropriate level for tests and full reporting of outcomes
☒	☐	Estimates of effect sizes (e.g. Cohen's d, Pearson's r), indicating how they were calculated

Our web collection on statistics for biologists contains articles on many of the points above.

Software and code

Policy information about availability of computer code

Data collection	No software was used for data collection.
Data analysis	R, TRACE, PRS-CS, LatentGOLD 5.1

For manuscripts utilizing custom algorithms or software that are central to the research but not yet described in published literature, software must be made available to editors and reviewers. We strongly encourage code deposition in a community repository (e.g. GitHub). See the Nature Portfolio guidelines for submitting code & software for further information.

Data

Policy information about availability of data

All manuscripts must include a data availability statement. This statement should provide the following information, where applicable:

- Accession codes, unique identifiers, or web links for publicly available datasets
- A description of any restrictions on data availability
- For clinical datasets or third party data, please ensure that the statement adheres to our policy

The data used in this manuscript from the Manchester Longitudinal Cognitive Ageing Cohort are not publicly available. However, they may be shared upon reasonable request and the completion of a Material Transfer Agreement.

Research involving human participants, their data, or biological material

Policy information about studies with human participants or human data. See also policy information about sex, gender (identity/presentation), and sexual orientation and race, ethnicity and racism.

Reporting on sex and gender	All analyses have considered sex as a covariate consistent with the language used for data collection.
Reporting on race, ethnicity, or other socially relevant groupings	Ancestry was determined from genetic data. Participants self-reported their race and ethnicity. Given the homogenous population, these were not included as covariates in analyses. However, principal components of ancestry were included as covariates in the genetic analyses.
Population characteristics	A total of 2,945 community-dwelling older adult participants had available meal timing data upon enrollment (baseline) and at least one additional repeat assessment. At baseline, participants had a mean age of 64.0 years (standard deviation (SD) = 6.6), 71.5% were female, and 83.3% were unemployed.
Recruitment	The University of Manchester Longitudinal Study of Cognition in Normal Healthy Old Age (UMLCHA) recruited 6,375 individuals aged 42-94 years old from Newcastle and Manchester, UK, starting in 1983 through 1993.
Ethics oversight	The study was approved by the University Research Ethics Committee at the University of Manchester (Ref: 2021-11274-17829), and all participants gave written informed consent prior to study procedures.

Note that full information on the approval of the study protocol must also be provided in the manuscript.

Field-specific reporting

Please select the one below that is the best fit for your research. If you are not sure, read the appropriate sections before making your selection.

☒ Life sciences ☐ Behavioural & social sciences ☐ Ecological, evolutionary & environmental sciences

For a reference copy of the document with all sections, see nature.com/documents/nr-reporting-summary-flat.pdf

Life sciences study design

All studies must disclose on these points even when the disclosure is negative.

Sample size	Participants who provided responses to all meal and sleep timing questions at baseline and had at least one additional meal timing response at any of the four subsequent occasions were included in the analysis. A total of 2,945 community-dwelling older adult participants had available meal timing data upon enrollment (baseline) and at least one additional repeat assessment.
Data exclusions	No data were excluded on the analysis. For time-dependent variables with missing data in later cycles, values were carried forward once from previous cycles using the last observation carried forward approach. If still missing after the first carry-forward, the data were treated as missing.
Replication	No replication was sought given the unique nature of the data included in the present analysis.
Randomization	As an observational study, no randomization was conducted.
Blinding	Given the observational nature of the study and the analysis of secondary outcomes in the present study, no blinding was necessary.

Reporting for specific materials, systems and methods

We require information from authors about some types of materials, experimental systems and methods used in many studies. Here, indicate whether each material, system or method listed is relevant to your study. If you are not sure if a list item applies to your research, read the appropriate section before selecting a response.

Materials & experimental systems

n/a	Involved in the study	
☒	☐	Antibodies
☒	☐	Eukaryotic cell lines
☒	☐	Palaeontology and archaeology
☒	☐	Animals and other organisms
☒	☐	Clinical data
☒	☐	Dual use research of concern
☒	☐	Plants

Methods

n/a	Involved in the study	
☒	☐	ChIP-seq
☒	☐	Flow cytometry
☒	☐	MRI-based neuroimaging

Plants

Seed stocks	*Report on the source of all seed stocks or other plant material used. If applicable, state the seed stock centre and catalogue number. If plant specimens were collected from the field, describe the collection location, date and sampling procedures.*
Novel plant genotypes	*Describe the methods by which all novel plant genotypes were produced. This includes those generated by transgenic approaches, gene editing, chemical/radiation-based mutagenesis and hybridization. For transgenic lines, describe the transformation method, the number of independent lines analyzed and the generation upon which experiments were performed. For gene-edited lines, describe the editor used, the endogenous sequence targeted for editing, the targeting guide RNA sequence (if applicable) and how the editor was applied.*
Authentication	*Describe any authentication procedures for each seed stock used or novel genotype generated. Describe any experiments used to assess the effect of a mutation and, where applicable, how potential secondary effects (e.g. second site T-DNA insertions, mosiacism, off-target gene editing) were examined.*
